# Antibiotic optimization in hospitalized children with non-severe community-acquired pneumonia: lessons from an antimicrobial stewardship intervention (2022–2024)

**DOI:** 10.3389/fped.2025.1660776

**Published:** 2025-11-04

**Authors:** Federica Attaianese, Roberto Privato, Carlotta Montagnani, Micol Stivala, Sandra Trapani, Luisa Galli, Giuseppe Indolfi

**Affiliations:** ^1^ Pediatric Unit, Meyer Children’s Hospital IRCCS, Florence, Italy; ^2^ Department of Health Science, University of Florence, Florence, Italy; ^3^ Pediatric Infectious Diseases Unit, Meyer Children’s Hospital IRCCS, Florence, Italy; ^4^ NEUROFARBA Department, University of Florence, Florence, Italy

**Keywords:** community-acquired pneumonia, children, antimicrobial stewardship, antibiotics, therapy duration

## Abstract

**Background and objectives:**

Community-acquired pneumonia (CAP) is a leading cause of hospitalization and antibiotic use in children. Despite guidelines recommending narrow-spectrum regimens and shorter treatment durations, prescribing practices remain inconsistent. This study assessed the impact of a newly implemented diagnostic and therapeutic clinical pathway (CP) as part of an antimicrobial stewardship (AMS) intervention in a tertiary care pediatric hospital.

**Methods:**

A single-center, retrospective observational study was conducted on children aged 28 days to 18 years hospitalized with non-severe, uncomplicated CAP from January 2022 to December 2024. The CP was implemented on January 1st, 2024. Antibiotic prescribing patterns, clinical outcomes, and predictors of short-course therapy (≤5 days) were compared between pre- and post-CP periods. Multivariate logistic regression identified predictors of intravenous (IV) therapy ≤48 h, total therapy ≤5 days, and ampicillin use as first-line agent.

**Results:**

The study included 263 CAP episodes in 250 children. Following the implementation of CP, the use of ampicillin as a first-line IV antibiotic significantly increased [19/99 (19%) vs. 1/164 (0.6%); *p* < 0.001]. A higher proportion of post-CP patients received IV antibiotics for ≤48 h [25/99 (25%) vs. 20/164 (12%); *p* = 0.006], reflecting an increased rate of early IV-to-oral switch. However, total antibiotic duration and hospital length of stay (LOS) remained unchanged. Viral detection in respiratory samples predicted antibiotic courses of ≤5 days.

**Conclusions:**

CP implementation improved adherence to evidence-based antibiotic prescribing, reduced broad-spectrum use, and increased early IV-to-oral transitions without compromising outcomes. However, unchanged therapy duration and LOS highlight the need for further AMS interventions, clinician education, and integration of viral and bacterial diagnostics to support optimal antibiotic use.

## Highlights

Implementing a clinical pathway for pediatric CAP in hospitalized patients improved antibiotic stewardship by increasing the use of ampicillin and reducing broad-spectrum antibiotic regimens. It also promoted earlier intravenous-to-oral switch without compromising clinical outcomes. However, the overall duration of antibiotic therapy, intravenous treatment, and hospital stay remained unchanged, highlighting areas for improvement.

## 1 Introduction

Community-acquired pneumonia (CAP) remains one of the leading causes of hospitalization and antimicrobial use in children worldwide. Despite progress in vaccination programs and diagnostic technologies, CAP remains a major clinical and public health challenge, contributing to considerable morbidity, healthcare costs, and the growing threat of antimicrobial resistance (AMR) (1–3). The inappropriate or prolonged use of broad-spectrum antibiotics exacerbates AMR and negatively affects patient outcomes, leading to extended hospital stays and a higher risk of adverse drug reactions (4–6).

National and international guidelines emphasize using narrow-spectrum antibiotics in children with uncomplicated CAP, especially in those who are previously healthy and vaccinated (7, 8). The choice of ampicillin or amoxicillin as first-line therapy for children without risk factors for resistant pathogens is widely recommended, given its effectiveness against common causative organisms such as *Streptococcus pneumoniae* and its favorable safety profile (7, 8). International guidelines recommend the use of high-dose, fractionated amoxicillin/ampicillin regimens to ensure efficacy also against strains with reduced susceptibility (7). In addition, recent scientific evidence has increasingly supported the non-inferiority of short-course antibiotic therapy, precisely five days of treatment, for children with mild-to-moderate, uncomplicated CAP (9–12). Multiple randomized controlled trials and systematic reviews have demonstrated that shorter regimens are equally effective as traditional longer courses (10–18). However, despite these findings, implementing narrow-spectrum and short-course therapy in routine clinical practice remains inconsistent, particularly in hospital settings, where variations in prescribing habits and clinical uncertainty often lead to unnecessarily prolonged treatment durations.

Antimicrobial stewardship (AMS) programs play a crucial role in optimizing antibiotic use, improving clinical outcomes, and addressing AMR (19–21). In January 2024, our tertiary-care pediatric university hospital, Meyer Children's Hospital IRCCS, introduced a diagnostic and therapeutic clinical pathway (CP) for CAP management as part of a broader AMS strategy. This protocol was specifically designed to align clinical practice with current evidence, encouraging shorter courses of antibiotic therapy and the use of first-line antibiotics when appropriate.

The present study provides a preliminary analysis of data collected before and after the CP implementation, focusing on antibiotic prescribing patterns, clinical outcomes, and predictors of optimal antibiotic use. Specifically, we aim to evaluate the feasibility and impact of adopting evidence-based short-course therapy in an inpatient pediatric setting, contributing to the growing literature on AMS in children.

## 2 Methods

### 2.1 Study design and inclusion criteria

We conducted a single-center observational and retrospective study. The Strengthening the Reporting of Observational Studies in Epidemiology (STROBE) guidelines were used to report the study (Supplementary Table S1).

The inclusion criteria were as follows: children aged between 28 days and 18 years at the date of infection, hospitalized at Meyer Children's Hospital IRCCS from January 1st 2022, to December 31st, 2024, diagnosed with non-severe and uncomplicated CAP. Patients who experienced separate infectious episodes, as defined by an interval of more than 60 days between them, were included twice. The study exclusion criteria encompass patients with cystic fibrosis or other chronic respiratory diseases (excluding asthma), those with immunodeficiencies or receiving immunosuppressive therapy, those with concomitant infections, individuals who are tracheostomized, and those with a diagnosis of aspiration pneumonia. Patients with incomplete information about treatment (duration and route) and those who received antibiotics for ≤48 h were also excluded. Potential participants were identified by screening the pediatric ward medical record database using the ninth edition of the International Classification of Diseases diagnostic codes (Supplementary Section 1.1). Demographic and clinical data, vaccination status, antibiotic type and duration, viral detection, and laboratory values were collected from electronic medical records. Details on the polymerase chain reaction (PCR) method used for viral detection on pharyngeal swab/blood samples are reported in the Supplementary Material (Supplementary Section 1.2).

### 2.2 Definitions

CAP was defined as an acute infection of the lung parenchyma acquired outside the hospital or other healthcare settings, clinically diagnosed by the presence of fever (≥38.5 °C) and at least one sign and one symptom among the following (8, 22):

• Signs: tachypnea, dyspnea, fine crackles, crepitations, reduced breath sounds.
• Symptoms: cough, sputum production, chest pain, loss of appetite.

The absence of the following local and systemic complications defined CAP as uncomplicated: parapneumonic effusion, empyema, necrotizing pneumonia, lung abscess, sepsis, septic shock, metastatic infection, multiorgan failure, acute respiratory distress syndrome, disseminated intravascular coagulation, and death (23).

The severity of CAP was defined, as illustrated in Supplementary Table S2 (24). Children who received less than two doses of the specific vaccine were considered unvaccinated for *Haemophilus influenzae* (8).

Narrow-spectrum and broad-spectrum regimens were defined following previous research (25). Amoxicillin or ampicillin alone was considered a narrow-spectrum regimen. Broad-spectrum antimicrobials were defined as β-lactam and β-lactamase inhibitor combinations, second- and third-generation cephalosporins, clindamycin, glycopeptides, fluoroquinolones, and macrolides. Therapeutic regimens, including at least one broad-spectrum prescription, were considered broad-spectrum despite their association with amoxicillin or ampicillin (25).

The duration of antibiotic therapy was calculated starting from the first day of effective antibiotic administration [either intravenous (IV) or oral] and continued by counting each calendar day of treatment until the last administered dose, including both inpatient and outpatient therapy when applicable. Total antibiotic duration was defined as the sum of IV and oral treatment periods.

Treatment failure was defined as either (13, 14, 25): (i) need for antibiotic treatment change because of no clinical response or clinical relapse, including a switch to IV therapy after initial oral therapy or switch from narrow-spectrum to broad-spectrum antimicrobials; (ii) hospital readmission for persistence or relapse of symptoms or for drug side effects (e.g., rash, diarrhea) within 30 days after completion of antibiotic treatment.

### 2.3 Antibiotic stewardship intervention

In 2023, a systematic literature review was conducted on the diagnosis and treatment of CAP in children to inform the development of a CP (Supplementary Section 1.3) for managing CAP by a multidisciplinary team comprising pediatricians, pediatric infectious disease specialists, pediatric pulmonologists, pediatric anesthetists, and pediatric surgeons. The pathway encompasses both severe and non-severe CAP, as well as uncomplicated and complicated cases. At the end of December 2023, an open meeting was held for all hospital physicians to present the CP and address any questions or concerns they may have. Additionally, a pocket-sized summary flowchart was distributed (Supplementary Material, “CAP flowchart”). The CP was officially implemented on January 1st, 2024. Since then, a semestral application monitoring program has been implemented using three key indicators for non-severe and uncomplicated CAP: (i) prescription of amoxicillin as 1st choice oral antibiotic therapy for mild-to-moderate, uncomplicated CAP; (ii) prescription of ampicillin as 1st choice IV antibiotic therapy for mild-to-moderate, uncomplicated CAP; (iii) a 5-day duration of therapy for mild-to-moderate, uncomplicated cases with a positive response to treatment. To promote adherence, the CP was incorporated into routine ward practice through multidisciplinary discussions, daily prescription reviews, and reinforcement during departmental meetings. Frontline providers received feedback both informally during clinical rounds and formally through periodic audit sessions. Although no formal re-implementation sessions were organized, the CP was subsequently integrated into the institutional set of clinical protocols, which helped support its visibility and sustainability beyond the initial implementation phase.

### 2.4 Statistical analysis

We described the demographic and clinical characteristics of the study population and compared therapeutic management between the pre- and post-CP periods. The primary outcome was to compare the proportion of CAP treated with ampicillin (if IV treatment was needed) or oral amoxicillin as the 1st choice between the two groups. The secondary outcomes were as follows: (i) the proportion of patients in each group receiving antibiotic therapy for ≤5 days; (ii) the hospital length of stay (LOS), compared between the two groups; (iii) the rate of treatment failure, compared between the two groups; (iv) predictors of IV antibiotic therapy duration ≤48 h in the overall sample; (v) predictors of total antibiotic therapy duration ≤5 days in the overall sample. Quantitative variables were portrayed as medians and interquartile ranges (IQR). Categorical variables were expressed as numbers (*n*) and percentages (%).

The Mann–Whitney *U* test was used to compare continuous-type patient characteristics. Statistical differences between categorical variables were analyzed using the *χ*^2^ or Fisher's exact test. Univariate and multivariate binary logistic regression was performed to identify predictors of IV antibiotic therapy ≤48 h, total antibiotic therapy ≤5 days, and use of ampicillin as first-choice antimicrobial. Variables with *p*-value ≤ 0.20 in the univariate analysis were included in the multivariate model, along with clinically or epidemiologically relevant variables. Hosmer and Lemeshow's test were used to assess the model's goodness of fit. Unadjusted and adjusted odds ratios, along with 95% confidence intervals (CI), were reported. All statistical tests were two-sided; a *p-value* of less than 0.05 was considered statistically significant. All data were analyzed using the SPSS statistical package, release 21.0 (SPSS, Inc., Chicago, Illinois).

## 3 Results

### 3.1 General cohort's characteristics

**Figure 1:**
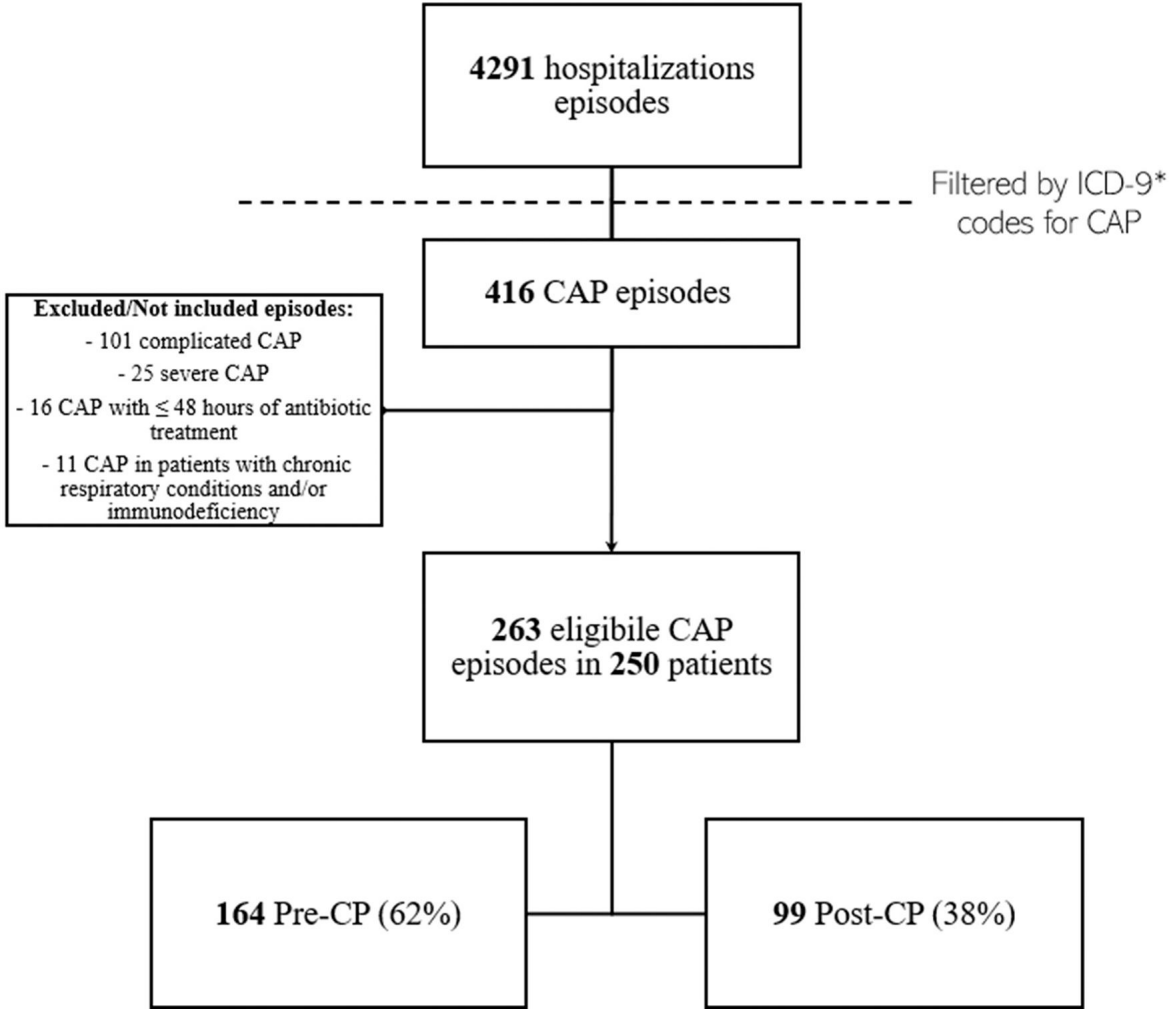
CAP episodes screening, identification, and inclusion flow-chart. *Reported in Supplementary Section 1.1. CAP, community acquired pneumonia; CP, diagnostic and therapeutic care pathway.

The retrospective chart review yielded 263 cases of CAP, corresponding to 250 patients (Figure 1). The average annual incidence of CAP was 62 cases per 1000 admissions. Characteristics of the overall population are summarized in Table 1. The median age was 4 years (IQR 2–8). Ninety-two episodes (35%) occurred in patients with comorbidities, with neurological/neuromuscular disorders (29/92, 32%) and multi-malformation syndromes (17/92, 19%) being the most represented. A history of suspected allergy to aminopenicillin was present in three cases (1%), while incomplete vaccination for *H. influenzae* was detected in 24 cases (9%). Oral antibiotic therapy at home before hospital admission has been administered in 60 cases (23%), with a median duration of 5 days [IQR (3–7)]. Concerning inpatient CAP management at admission, 241 CAP episodes (92%) were initially managed with IV antibiotics, and among these, 20 (8%) were treated with ampicillin; 22 episodes (9%) were treated with oral antibiotics without a switch to the IV route during hospitalization. Of these, three (14%) received amoxicillin. Forty-five cases (17%) were treated with IV antibiotics for 48 h or less, and 41 (16%) received parenteral antibiotics exclusively. The total antibiotic course was shorter in patients who exclusively received oral therapy compared to the rest of the sample [7 days, IQR (5.5–10) vs. 10 days, IQR (9–13), *p* < 0.001]. Cases switched to oral therapy after initial IV administration had a shorter hospital LOS [6 days, IQR (5–8) vs. 7 days, IQR (5–13), *p* *=* 0.024] and a shorter duration of IV therapy [5 days, IQR (3–7) vs. 6 days, IQR (5–10), *p* = 0.001] compared to those who received IV therapy only. On the other hand, they had longer antibiotic duration [10.5 days, IQR (10–13) vs. 6 days, IQR (5–10), *p* < 0.001].

**Table 1:** Demographic and clinical characteristics of children hospitalized for CAP.

Variable	Total	Pre-CP	Post-CP	*p*
	(*n* = 263)	(*n* = 164)	(*n* = 99)	
Age in years [median (IQR)]	4 (2–8)	4 (2–7)	4 (3–9)	0.30
Males [*n*; (%)]	150; (57)	94; (57)	56; (57)	0.90
Comorbidities [*n*; (%)]	92; (35)	50; (30)	42; (42)	**0** **.** **049**
History of reaction to aminopenicillins [*n*; (%)]	3; (1)	3; (2)	0; (0)	0.29
Vaccinated for Haemophilus influenza [*n*; (%)]	239; (91)	149; (93)	90; (91)	0.52
Oxygen therapy [*n*; (%)]	135; (51)	82; (50)	53; (53)	0.58
Positive blood/pharyngeal swab viral PCR [*n*; (%)]	76; (29)	46; (28)	30; (30)	0.70
WBC×10^3^/μL [median (IQR)]	13.5; (10.3–19.4)	13.9 (10.6–19.7)	13; (8–19.1)	0.33
CRP mg/dL [median (IQR)]	5; (1.8–13.8)	6; (2.3–14)	2.7 (1–12)	**0** **.** **02**
Oral antibiotic started at home [*n*; (%)]	60; (23)	25; (15)	35; (35)	**<0** **.** **001**
Initial IV therapy [*n*; (%)]	241; (92)	151; (92)	90; (91)	0.99
Ampicillin as first-choice IV therapy [*n*; (%)]	20; (8)	1; (0.6)	19; (19)	**<0** **.** **001**
IV therapy for ≤48 h [*n*; (%)]	45; (17)	20; (12)	25; (25)	**0** **.** **006**
Oral amoxicillin [*n*; (%)]	13; (5)	8; (5)	5; (5)	0.95
Broad-spectrum antibiotics (either oral or IV) [*n*; (%)]	232; (88)	155; (94)	77; (78)	**<0** **.** **001**
IV therapy duration [median (IQR)]	5 (4–7)	5 (4–7)	5 (3–8)	0.90
Oral therapy duration [median (IQR)]	5 (4–7)	5 (4–7)	5 (3–7)	0.69
Total therapy duration [median (IQR)]	10 (9–13)	10 (9–12)	10 (7–14)	0.80
Hospital LOS [median (IQR)]	6 (5–8)	6 (57)	7 (5–9)	0.06
Treatment failure [*n*; (%)]	6 (2)	2 (1)	4 (4)	0.14
Need for treatment change	-	-	-	
Hospital readmission	6 (2)	2 (1)	4 (3.5)	

CP: diagnostic and therapeutic clinical pathway; IQR (interquartile range); PCR: polymerase chain reaction; IV: intravenous; LOS: length-of-stay; WBC: total white blood cell count; CRP: C-reactive protein.

Bold values denote statistically significant differences (*p* < 0.05).

Children with comorbidities had a longer course of IV therapy (6 days, IQR 4–9, vs. 5 days IQR 4–7, *p* = 0.012), and a longer hospital LOS (7 days, IQR 5–11 vs. 6 days, IQR 5–7, *p* < 0.001). Previously started antibiotics and virus detection on pharyngeal swab/blood by PCR testing predicted a ≤5-day antibiotic course in the overall sample (Table 2). In our cohort, 13.2% of children with a positive viral test discontinued antibiotics within 48 h compared with 2.7% of those without viral detection. No adverse reactions to antibiotic therapy were reported across the observation period.

**Table 2:** Univariate and multivariate predictors of total antibiotic therapy ≤5 days in children hospitalized for CAP.

Variable	Univariate analysis			Multivariate analysis		
	uOR	CI 95%	*p*	aOR	CI 95%	*p*
Age	0.9	0.8–1.1	0.60			
Virus detection	4.8	2–12	**<0** **.** **001**	5.8	2.2–15.6	**<0** **.** **001**
CP implementation	1.7	0.7–4.3	0.25			
Comorbidities	0.6	0.2–1.7	0.69			
Previous episodes	1.1	0.7–1.7	0.53			
Oral therapy started at home	3.7	1.5–9.2	**0** **.** **004**	4.7	1.8–12.5	**0** **.** **002**
WBC at admission	1	0.9–1.1	0.25			
CRP at admission	0.9	0.8–1.1	0.66			

CP, diagnostic and therapeutic clinical pathway; WBC, total white blood cell count; CRP, C-reactive protein; uOR, unadjusted odds ratio; aOR, adjusted odds ratio; CI, confidence interval; p, *p*-value.

Bold values denote statistically significant differences (*p* < 0.05).

### 3.2 Pre- and post-CP CAP management

The comparison of the demographic and clinical characteristics between the pre-CP and the post-CP groups is summarized in Table 1. The post-CP sample showed an increased use of ampicillin as a first-choice IV antimicrobial (19% vs. 0.6%, *p* < 0.001) (Tables 1, 3), along with a higher rate of children treated with IV antimicrobials for ≤48 h (25% vs. 12%, *p* = 0.006). According to the multivariate logistic regression analysis (Table 4), CP implementation was the only variable predictive of IV therapy course ≤48 h in the overall sample. The prescription rate of broad-spectrum antibiotics was lower in the post-CP (94% vs. 78%, *p* < 0.001). In the post-CP group, two cases involved switching from a broad-spectrum to a narrow-spectrum IV therapy regimen, whereas no such cases occurred in the pre-CP group. The rate of cases treated for ≤5 days was similar between the groups (9% vs. 13%, *p* = 0.23), as were the median durations of IV therapy and hospital LOS (Table 1). There was no difference in treatment failure rates between the groups (Table 1).

**Table 3:** Univariate and multivariate predictors of use of ampicillin as first choice in children hospitalized for CAP.

Variable	Univariate analysis			Multivariate analysis		
	uOR	CI 95%	*p*	aOR	CI 95%	*p*
Age	1.1	0.9–1.3	0.37			
CP implementation	29	4–150	**0**.**001**	31	3–180	**0**.**004**
Comorbidities	0.8	0.2–3	0.70			
Previous episodes	1.3	0.7–2.2	0.35			
Oral therapy started at home	0.8	0.1–3.8	0.77			
WBC at admission	0.9	0.8–1	0.91			
CRP at admission	1	0.9–1.1	0.80			
Season	0.7	0.2–2	0.55	1	0.3–3	0.94

CP, diagnostic and therapeutic care pathway; WBC, total white blood cell count; CRP, C-reactive protein; uOR, unadjusted odds ratio; aOR, adjusted odds ratio; CI, confidence interval; p, *p*-value.

Bold values denote statistically significant differences (*p* < 0.05).

**Table 4:** Univariate and multivariate predictors of intravenous antibiotic therapy for ≤48 h in children hospitalized for CAP.

Variable	Univariate analysis			Multivariate analysis		
	uOR	CI 95%	*p*	aOR	CI 95%	*p*
Age	0.9	0.8–1	0.82			
Virus detection	2	0.9–4.3	0.06	1.9	0.9–4.2	0.08
CP implementation	2.3	1–5	**0**.**03**	2.2	1–4.7	**0**.**04**
Comorbidities	1.1	0.5–2.4	0.72			
Previous episodes	0.9	0.6–1.4	0.72			
Oral therapy started at home	1.6	0.7–3.6	0.21			
WBC at admission	1	0.9–1.1	0.5			
CRP at admission	1	0.9–1.1	0.63			
Season	0.8	0.6–1.1	0.22	0.9	0.6–1.3	0.59

CP, diagnostic and therapeutic care pathway; WBC, total white blood cell count; CRP, C-reactive protein; uOR, unadjusted odds ratio; aOR, adjusted odds ratio; CI, confidence interval; p, *p*-value.

Bold values denote statistically significant differences (*p* < 0.05).

## 4 Discussion

### 4.1 Main findings

This study comprehensively evaluates the impact of CP implementation on managing pediatric CAP in a tertiary-care hospital setting. The CP introduction led to a marked improvement in antibiotic prescribing practices, evidenced by an outstanding increase in the use of ampicillin as the first-line IV antibiotic for mild-to-moderate, uncomplicated CAP and a parallel reduction in broad-spectrum antibiotic use. Notably, these improvements occurred without significant differences in hospital LOS or treatment failure rates, reinforcing the safety and feasibility of evidence-based antimicrobial stewardship strategies. Of note, pathway implementation did not affect the overall rate of antibiotic prescriptions, as all included patients met criteria for CAP requiring therapy.

Despite these positive outcomes, the proportion of patients treated for ≤5 days remained unchanged between the pre- and post-CP periods (9% vs. 13%, *p* *=* 0.23). Furthermore, while the CP facilitated shorter IV therapy in some cases, the median duration of IV treatment and the total antibiotic course remained unaffected, suggesting areas that require further intervention.

### 4.2 Interpretation and previous findings

Clinical pathways have been shown to improve AMS in pediatric CAP management (25), and our findings align with this evidence. The observed reduction in broad-spectrum antibiotic use and increased reliance on ampicillin are consistent with best-practice guidelines emphasizing the narrow-spectrum antibiotic regimen use to mitigate resistance risks (4). The higher rate of children receiving IV antibiotics for ≤48 h in the post-CP group highlights a shift toward early oral therapy transitions, a practice associated with reduced costs, shorter hospital LOS, and decreased complications related to IV catheters (5, 6, 26). However, the CP implementation did not affect the median duration of IV therapy (Table 1). Several factors may explain this result. Although the post-CP group included a higher proportion of patients with underlying comorbidities (Table 1), this factor was not associated with IV therapy duration in the univariate and multivariate analyses. Nevertheless, it is plausible that in clinical practice the presence of complex medical conditions influenced physicians' attitudes toward antibiotic management, contributing to a more cautious approach in some cases. Notably, more than 30% of children with comorbidities had neurological conditions, which are typically associated with difficulties in oral administration. Overall, patients with comorbidities received significantly longer courses of antibiotics, suggesting an enhanced concern for disease progression when managing children with baseline complex medical conditions. Moreover, aspiration pneumonia is a significant cause of mortality in this patient group (27), and challenges in the differential diagnosis may result in extended antibiotic therapy. In addition, some contextual factors may explain the limited impact of the intervention on prescribing practices, such as the availability of diagnostic tools, previous antibiotic exposure, and variability in implementation across wards. During the preparatory phase, discussions focused on the choice of first-line antibiotic. Ampicillin was recommended in line with stewardship principles and guideline-based evidence, while ceftriaxone was reserved for defined clinical scenarios such as non-vaccinated children, complicated infections, or treatment failure. This distinction reflects the rationale of promoting narrow-spectrum therapy whenever possible, while ensuring adequate coverage and feasibility when broader-spectrum agents are clinically justified.

Nevertheless, high-quality evidence regarding optimal management strategies in this subset of patients remains limited. Another reason for the reluctance to reduce IV courses presumably lies in the outdated belief that the IV route is inherently more effective than the oral one and that the inpatient setting should imply the use of IV therapy. However, Cotter et al. (28) demonstrated that children with CAP receiving initial oral antibiotics had an 8% reduction of hospital LOS and a 14% reduction in costs, without significant differences in clinical outcomes compared to children treated with the IV route. They also showed that patients treated with initial oral antimicrobials were rarely transitioned to IV administration, suggesting the role of a “route momentum” phenomenon in which physicians tend to continue the same route that was started. Accordingly, 22 patients in our sample were initially treated with the oral route, and none were subsequently switched to IV. Moreover, the overall antibiotic course was shorter in children who exclusively received oral therapy [7 days, IQR (5.5–10) vs. 10 days, IQR (9–13), *p* < 0.001]. Furthermore, cases transitioned to oral therapy after initial IV administration experienced shorter IV courses and hospital LOS compared to those treated exclusively with parenteral therapy. Otherwise, the total duration of antibiotic therapy was longer in these patients when compared to those treated exclusively with IV therapy, possibly reflecting greater clinical confidence in the adequacy of IV treatment alone.

According to multivariate analysis, the positive result of pharyngeal swab/blood viral PCR testing was predictive of a total antibiotic duration of ≤5 days (Table 2). This result is not surprising, as identifying a consistent etiology may increase physicians' confidence in discontinuing antibiotics. Evidence suggests that viral PCR testing may positively affect clinical decision-making and antibiotic stewardship. Indeed, previous literature has shown that the positivity of the respiratory viral panel PCR correlates with a shorter course of antibiotics in febrile infants (29). Moreover, Galetto-Lacour et al. (30) found that virus detection through blood PCR is a reliable predictor of the absence of severe bacterial infections. Further studies are needed to clarify the potential role of different specimen PCR testing in guiding CAP therapy. Moreover, standardizing viral PCR testing and conducting comprehensive cost-effectiveness analyses are essential to optimize its clinical use.

Despite these improvements, the overall duration of antibiotic therapy remained constant, underscoring the need for ongoing education and further interventions to fully optimize prescribing practices.

Although our study did not directly assess AMR outcomes, reducing the unnecessary use of broad-spectrum and/or protracted antibiotic regimens is widely recognized as a key strategy to mitigate the emergence of AMR. Previous studies have demonstrated that hospital-based antimicrobial stewardship interventions are associated with a decrease in resistance patterns over time (14, 16, 21, 26).

## 5 Limitations

This study has several limitations. First, as a retrospective analysis, it is subject to reporting inaccuracies and missing data. Second, the lack of follow-up prevents us from identifying recurrent cases managed in secondary or primary care settings. However, our institution is the only tertiary pediatric hospital in Tuscany, serving as a referral center for complex infectious and respiratory diseases, which strengthens the generalizability of our findings. Another limitation is that the association between pre-hospital antibiotic exposure and shorter overall treatment duration is difficult to interpret, as no information was available on the agents or regimens administered at home. Finally, delays and limited access to viral PCR results likely reduced the pathway's impact on treatment duration and introduced bias in prescribing patterns. Concerns regarding the cost of multiplex respiratory viral PCR panels may have discouraged their systematic use by clinicians. More broadly, cost–benefit analyses are essential to define the potential role of these tests as an integrated component of a clinical CP.

## 6 Conclusion

Implementing a diagnostic and therapeutic CP significantly improved antibiotic prescribing practices for pediatric CAP, notably increasing the use of ampicillin and reducing the use of broad-spectrum antibiotic regimens. The CP also facilitated a higher rate of early transitions from IV to oral therapy without compromising clinical outcomes. However, the overall duration of antibiotic therapy, IV treatment, and hospital LOS remained unchanged, highlighting areas for further improvement. Future efforts should focus on clinician education, addressing persistent misconceptions about the efficacy of oral therapy, and standardizing definitions of treatment failure to better inform clinical decisions. Moreover, integrating viral and bacterial PCR testing into routine clinical practice may further enhance antimicrobial stewardship by enabling more targeted and shortened antibiotic regimens. Narrow-spectrum and/or short-course antibiotic regimens not only offer better tolerability and reduced healthcare costs but also play a key role in limiting the emergence of AMR, a major global health threat.

## Data Availability

The raw data supporting the conclusions of this article will be made available by the authors, without undue reservation.

## References

[1] Walker CLF Rudan I Liu L Nair H Theodoratou E Bhutta ZA Global burden of childhood pneumonia and diarrhoea. Lancet. (2013) 381(9875):1405–16. 10.1016/S0140-6736(13)60222-6 23582727 PMC7159282

[2] Zar HJ Ferkol TW. The global burden of respiratory disease-impact on child health: the global burden of respiratory disease. Pediatr Pulmonol. (2014) 49(5):430–4. 10.1002/ppul.23030 24610581

[3] World Health Statistics. World Health Organization (2024). Available online at: https://iris.who.int/bitstream/handle/10665/376869/9789240094703-eng.pdf (Accessed May 30, 2025).

[4] Huttner A Harbarth S Carlet J Cosgrove S Goossens H Holmes A Antimicrobial resistance: a global view from the 2013 world healthcare-associated infections forum. Antimicrob Resist Infect Control. (2013) 2(1):31. 10.1186/2047-2994-2-31 24237856 PMC4131211

[5] Jumani K Advani S Reich NG Gosey L Milstone AM. Risk factors for peripherally inserted central venous catheter complications in children. JAMA Pediatr. (2013) 167(5):429. 10.1001/jamapediatrics.2013.775 23549677 PMC3647026

[6] Christensen EW Spaulding AB Pomputius WF Grapentine SP. Effects of hospital practice patterns for antibiotic administration for pneumonia on hospital lengths of stay and costs. J Pediatr Infect Dis Soc. (2019) 8(2):115–21. 10.1093/jpids/piy003 29438527

[7] Bradley JS Byington CL Shah SS Alverson B Carter ER Harrison C The management of community-acquired pneumonia in infants and children older than 3 months of age: clinical practice guidelines by the pediatric infectious diseases society and the infectious diseases society of America. Clin Infect Dis. (2011) 53(7):e25–76. 10.1093/cid/cir531 21880587 PMC7107838

[8] Donà D Brigadoi G Grandinetti R Pedretti L Boscarino G Barbieri E Treatment of mild to moderate community-acquired pneumonia in previously healthy children: an Italian intersociety consensus (SIPPS-SIP-SITIP-FIMP-SIAIP-SIMRI-FIMMG-SIMG). Ital J Pediatr. (2024) 50(1):217. 10.1186/s13052-024-01786-8 39427174 PMC11491012

[9] Shapiro DJ Hall M Lipsett SC Hersh AL Ambroggio L Shah SS Short- versus prolonged-duration antibiotics for outpatient pneumonia in children. J Pediatr. (2021) 234:205–211.e1. 10.1016/j.jpeds.2021.03.017 33745996

[10] Li Q Zhou Q Florez ID Mathew JL Shang L Zhang G Short-course vs long-course antibiotic therapy for children with nonsevere community-acquired pneumonia: a systematic review and meta-analysis. JAMA Pediatr. (2022) 176(12):1199. 10.1001/jamapediatrics.2022.4123 36374480 PMC9664370

[11] Kuitunen I Jääskeläinen J Korppi M Renko M. Antibiotic treatment duration for community-acquired pneumonia in outpatient children in high-income countries—a systematic review and meta-analysis. Clin Infect Dis. (2023) 76(3):e1123–8. 10.1093/cid/ciac374 35579504 PMC9907524

[12] Gao Y Liu M Yang K Zhao Y Tian J Pernica JM Shorter versus longer-term antibiotic treatments for community-acquired pneumonia in children: a meta-analysis. Pediatrics. (2023) 151(6):e2022060097. 10.1542/peds.2022-060097 37226686

[13] Greenberg D Givon-Lavi N Sadaka Y Ben-Shimol S Bar-Ziv J Dagan R. Short-course antibiotic treatment for community-acquired alveolar pneumonia in ambulatory children: a double-blind, randomized, placebo-controlled trial. Pediatr Infect Dis J. (2014) 33(2):136–42. 10.1097/INF.0000000000000023 23989106

[14] Barratt S Bielicki JA Dunn D Faust SN Finn A Harper L Amoxicillin duration and dose for community-acquired pneumonia in children: the CAP-IT factorial non-inferiority RCT. Health Technol Assess. (2021) 25(60):1–72. 10.3310/hta25600 34738518

[15] Pernica JM Harman S Kam AJ Carciumaru R Vanniyasingam T Crawford T Short-course antimicrobial therapy for pediatric community-acquired pneumonia: the SAFER randomized clinical trial. JAMA Pediatr. (2021) 175(5):475. 10.1001/jamapediatrics.2020.6735 33683325 PMC7941245

[16] Bielicki JA Stöhr W Barratt S Dunn D Naufal N Roland D Effect of amoxicillin dose and treatment duration on the need for antibiotic re-treatment in children with community-acquired pneumonia: the CAP-IT randomized clinical trial. JAMA. (2021) 326(17):1713. 10.1001/jama.2021.17843 34726708 PMC8564579

[17] Williams DJ Creech CB Walter EB Martin JM Gerber JS Newland JG Short- vs. standard-course outpatient antibiotic therapy for community-acquired pneumonia in children: the SCOUT-CAP randomized clinical trial. JAMA Pediatr. (2022) 176(3):253. 10.1001/jamapediatrics.2021.5547 35040920 PMC8767493

[18] McCallum GB Fong SM Grimwood K Nathan AM Byrnes CA Ooi MH Extended versus standard antibiotic course duration in children <5 years of age hospitalized with community-acquired pneumonia in high-risk settings: four-week outcomes of a multicenter, double-blind, parallel, superiority randomized controlled trial. Pediatr Infect Dis J. (2022) 41(7):549–55. 10.1097/INF.0000000000003558 35476706

[19] Gerber JS Kronman MP Ross RK Hersh AL Newland JG Metjian TA Identifying targets for antimicrobial stewardship in children’s hospitals. Infect Control Hosp Epidemiol. (2013) 34(12):1252–8. 10.1086/673982 24225609

[20] Gerber JS Jackson MA Tamma PD Zaoutis TE Maldonado YA, Committee on Infectious Diseases, Pediatric Infectious Diseases Society. Antibiotic stewardship in pediatrics. Pediatrics. (2021) 147(1):e2020040295. 10.1542/peds.2020-040295 33372120

[21] McMullan B Bryant PA Duffy E Bielicki J De Cock P Science M Multinational consensus antimicrobial stewardship recommendations for children managed in hospital settings. Lancet Infect Dis. (2023) 23(6):e199–207. 10.1016/S1473-3099(22)00726-5 36566768

[22] McIntosh K. Community-acquired pneumonia in children. N Engl J Med. (2002) 346(6):429–37. 10.1056/NEJMra011994 11832532

[23] De Benedictis FM Kerem E Chang AB Colin AA Zar HJ Bush A. Complicated pneumonia in children. Lancet. (2020) 396(10253):786–98. 10.1016/S0140-6736(20)31550-6 32919518

[24] Harris M Clark J Coote N Fletcher P Harnden A McKean M British thoracic society guidelines for the management of community acquired pneumonia in children: update 2011. Thorax. (2011) 66(Suppl 2):ii1–23. 10.1136/thoraxjnl-2011-200598 21903691

[25] Donà D Zingarella S Gastaldi A Lundin R Perilongo G Frigo AC Effects of clinical pathway implementation on antibiotic prescriptions for pediatric community-acquired pneumonia. PLoS One. (2018) 13(2):e0193581. 10.1371/journal.pone.0193581 29489898 PMC5831636

[26] McMullan BJ Andresen D Blyth CC Avent ML Bowen AC Britton PN Antibiotic duration and timing of the switch from intravenous to oral route for bacterial infections in children: systematic review and guidelines. Lancet Infect Dis. (2016) 16(8):e139–52. 10.1016/S1473-3099(16)30024-X 27321363

[27] Hirsch AW Monuteaux MC Fruchtman G Bachur RG Neuman MI. Characteristics of children hospitalized with aspiration pneumonia. Hosp Pediatr. (2016) 6(11):659–66. 10.1542/hpeds.2016-0064 27803071

[28] Cotter JM Zaniletti I Williams DJ Ramgopal S Fritz CQ Taft M Association between initial antibiotic route and outcomes for children hospitalized with pneumonia. J Hosp Med. (2024) 20(3):238–247. 10.1002/jhm.13382 39385410 PMC11875966

[29] Foppiano Palacios C Lemmon E Donohue KE Sutherland M Campbell J. Antibiotic use and respiratory viral PCR testing among pediatric patients with nosocomial fever. Cureus. (2023) 15(4):e37759. 10.7759/cureus.37759 37214055 PMC10193774

[30] Galetto-Lacour A Cordey S Papis S Mardegan C Luterbacher F Combescure C Viremia as a predictor of absence of serious bacterial infection in children with fever without source. Eur J Pediatr. (2023) 182(2):941–7. 10.1007/s00431-022-04690-7 36399200 PMC9672567

