# Supplementary material for: Antibiotic optimization in hospitalized children with non-severe community-acquired pneumonia: lessons from an antimicrobial stewardship intervention (2022–2024)
Source: Front Pediatr. 2025 Nov 4;13:1660776. doi: 10.3389/fped.2025.1660776 (PMC12623335; doi:10.3389/fped.2025.1660776)
Supplement: Supplementary file 1 [file Supplementaryfile1.docx]

**Supplementary Material**

**- Supplementary Table S1. STROBE Checklist**

**-** **Clinical chart review**

**- Polymerase chain reaction testing**

**- Clinical pathway**

**- CAP severity assessment criteria**

1. **METHODS**

**Supplementary Table S1. STROBE Checklist**

|  | Item No | Recommendation |
| --- | --- | --- |
| **Title and abstract** | 1 | (*a*) Indicate the study’s design with a commonly used term in the title or the abstract |
|  |  | (*b*) Provide in the abstract an informative and balanced summary of what was done and what was found |
| Introduction | | |
| Background/rationale | 2 | Explain the scientific background and rationale for the investigation being reported |
| Objectives | 3 | State specific objectives, including any prespecified hypotheses |
| Methods | | |
| Study design | 4 | Present key elements of study design early in the paper |
| Setting | 5 | Describe the setting, locations, and relevant dates, including periods of recruitment, exposure, follow-up, and data collection |
| Participants | 6 | (*a*) Give the eligibility criteria, and the sources and methods of selection of participants. Describe methods of follow-up |
|  |  | (*b*) For matched studies, give matching criteria and number of exposed and unexposed |
| Variables | 7 | Clearly define all outcomes, exposures, predictors, potential confounders, and effect modifiers. Give diagnostic criteria, if applicable |
| Data sources/ measurement | 8* | For each variable of interest, give sources of data and details of methods of assessment (measurement). Describe comparability of assessment methods if there is more than one group |
| Bias | 9 | Describe any efforts to address potential sources of bias |
| Study size | 10 | Explain how the study size was arrived at |
| Quantitative variables | 11 | Explain how quantitative variables were handled in the analyses. If applicable, describe which groupings were chosen and why |
| Statistical methods | 12 | (*a*) Describe all statistical methods, including those used to control for confounding |
|  |  | (*b*) Describe any methods used to examine subgroups and interactions |
|  |  | (*c*) Explain how missing data were addressed |
|  |  | (*d*) If applicable, explain how loss to follow-up was addressed |
|  |  | (*e*) Describe any sensitivity analyses |
| Results | | |
| Participants | 13* | (a) Report numbers of individuals at each stage of study—e.g. numbers potentially eligible, examined for eligibility, confirmed eligible, included in the study, completing follow-up, and analysed |
|  |  | (b) Give reasons for non-participation at each stage |
|  |  | (c) Consider use of a flow diagram |
| Descriptive data | 14* | (a) Give characteristics of study participants (eg demographic, clinical, social) and information on exposures and potential confounders |
|  |  | (b) Indicate number of participants with missing data for each variable of interest |
|  |  | (c) Summarize follow-up time (e.g, average and total amount) |
| Outcome data | 15* | Report numbers of outcome events or summary measures over time |
| Main results | 16 | (*a*) Give unadjusted estimates and, if applicable, confounder-adjusted estimates and their precision (e.g., 95% confidence interval). Make clear which confounders were adjusted for and why they were included |
|  |  | (*b*) Report category boundaries when continuous variables were categorized |
|  |  | (*c*) If relevant, consider translating estimates of relative risk into absolute risk for a meaningful time period |
| Other analyses | 17 | Report other analyses done—e.g. analyses of subgroups and interactions, and sensitivity analyses |
| Discussion | | |
| Key results | 18 | Summarise key results with reference to study objectives |
| Limitations | 19 | Discuss limitations of the study, taking into account sources of potential bias or imprecision. Discuss both direction and magnitude of any potential bias |
| Interpretation | 20 | Give a cautious overall interpretation of results considering objectives, limitations, multiplicity of analyses, results from similar studies, and other relevant evidence |
| Generalisability | 21 | Discuss the generalisability (external validity) of the study results |
| Other information | | |
| Funding | 22 | Give the source of funding and the role of the funders for the present study and, if applicable, for the original study on which the present article is based |

*Give information separately for exposed and unexposed groups.

**1.1 Clinical chart review**

The pediatric ward database was screened for all children presenting with non-severe and uncomplicated CAP from January 1^st^, 2022, to June 30^th^, 2024. We examined the outpatient clinic records for all instances of specific and non-specific codes related to CAP as per the International Classification of Disease, Ninth Revision (ICD-9). These included: 4829 for bacterial pneumonia, 481 for pneumococcal pneumonia, 486 for unspecified pneumonia, 5109 for pneumonia with effusion, 485 for bronchopneumonia, 4821 for pseudomonas pneumonia, 4822 for *Haemophilus pneumonia*, 48231 for group A *Streptococcus pyogenes* pneumonia, 48241 for *Staphylococcus aureus* pneumonia, 48281 for anaerobic pneumonia, 48289 for pneumonia caused by other bacteria, 4870 for influenza with pneumonia, and 5130 for lung abscess.

**1.2** **Polymerase chain reaction testing**

Nasopharyngeal swab PCR testing for SARS-CoV-2 was performed at the discretion of the attending physician. Testing for other respiratory viruses, using a separate multiplex panel, was also performed at the physician’s discretion. The panel of respiratory viruses included respiratory syncytial virus, Influenza virus (A, B), Parainfluenza virus (serotype 1, 2, 3), Adenovirus, Bocavirus, Metapneumovirus and Rhinovirus. Additional tests, including PCR for parechovirus, enterovirus, Epstein-Barr virus (EBV), and *Mycoplasma pneumoniae* were performed at the discretion of the attending physician. Blood samples were occasionally tested for EBV, enterovirus, and parechovirus as per the physician's decision based on the clinical presentation. Laboratory methods for PCR testing were those previously described by Lodi et al. (1).

**1.3 Clinical pathway**

**1.3.1 Diagnosis and Severity Assessment**

| **Domain** | **Criteria** |
| --- | --- |
| **Diagnosis** | Fever ≥38.5°C **AND** ≥1 symptom (cough, chest pain, anorexia) **AND** ≥1 sign (tachypnea, crackles, reduced breath sounds, bronchial breath sounds). |
| **Imaging** | CXR only if hospitalized, severe respiratory distress, or suspected complications. Ultrasound for pleural effusion. |
| **Laboratory** | CBC, CRP, PCT, blood culture before antibiotics (if febrile)  Respiratory viral PCR on nasal swab. |
| **Severity** | *Mild*: no distress, SpO₂ >92% RA, no sepsis.  *Moderate*: distress, SpO₂ <92%, need for HFNC <40% FiO₂.  *Severe*: respiratory failure requiring ventilation, shock/sepsis, altered perfusion, very high RR for age. |
|  |  |

**1.3.2 Admission Criteria**

- Age <4 months.
- Severe tachypnea (RR >70 if <12 mo, RR >50 if >12 mo).
- SpO₂ <92% in room air.
- Shock/sepsis, altered perfusion, or severe distress.
- Inability to feed or persistent vomiting.
- Relevant comorbidities.
- Complicated pneumonia.
- Failure of home therapy after 48–72h.

**1.3.3 Antibiotic Therapy***

| **Setting** | **First-line** | **Alternative / Second-line** |
| --- | --- | --- |
| **Outpatient (mild, non-complicated)** | Amoxicillin 90 mg/kg/day PO (3 doses).  If not Hib-vaccinated → Amoxicillin-clavulanate. | If allergy: Cefpodoxime, Levofloxacin, Clindamycin, Azithromycin. |
| **Hospitalized, non-complicated** | Ampicillin 200 mg/kg/day IV q6h.  If not Hib-vaccinated → Ampicillin-sulbactam or Ceftriaxone. | If failure at 48h → Ceftriaxone or Cefotaxime ± Azithromycin (>5 yrs). |
| **Complicated CAP** | Ceftriaxone 75–100 mg/kg/day + Clindamycin 45 mg/kg/day. | Severe sepsis: Ceftriaxone + Vancomycin ± Azithromycin (if atypical suspected). |
| **Duration** | 5 days (mild CAP); 10 days (moderate/severe uncomplicated); 2–4 weeks (complicated). |  |
| **Switch to oral** | As soon as clinical improvement and oral tolerance are achieved. |  |

*When a viral pathogen is detected and the clinical evolution is consistent with viral pneumonia, empiric antibiotic therapy was discontinued within 24–48 hours.

**1.3.4 Antiviral Therapy**

- **Oseltamivir** indicated for hospitalized children with influenza or at high risk of complications.
- Dosing: <10 kg → 3 mg/kg BID; 10–15 kg → 30 mg BID; 15–23 kg → 45 mg BID; 23–40 kg → 60 mg BID; >40 kg → 75 mg BID.

**1.3.5 Discharge and Follow-up**

**Discharge criteria**

- Afebrile for ≥24h.
- SpO₂ >92% RA.
- Clinical improvement and stable vital signs.
- Adequate oral intake and family compliance.
- Drain removed for ≥24h without deterioration (if applicable).

**Follow-up**

- None for uncomplicated CAP with full recovery.
- Complicated CAP: follow-up with infectious diseases, pneumology, and surgery as appropriate.

**Table S2. CAP severity assessment criteria.**

|  | **Mild to moderate** | **Severe** |
| --- | --- | --- |
| **Infants** | Temperature <38.5 C  Respiratory rate <70 breaths/min  Mild recession | Temperature >38.5 C  Respiratory rate >=70 breaths/min  Moderate to severe recession  Nasal flaring  Cyanosis  Intermittent apnea  Grunting respiration  Not feeding  Tachycardia*  Capillary refill time >2s |
| **Older children** | Temperature <38.5 C  Respiratory rate <50 breaths/min  Mild breathlessness  No vomiting | Temperature >38.5 C  Respiratory rate >=50 breaths/min  Severe difficulty in breathing  Nasal flaring  Cyanosis Grunting respiration  Signs of dehydration  Tachycardia*  Capillary refill time >2s |

*Values to define tachycardia vary with age and with temperature.

**REFERENCES**

1. Lodi L, Catamerò F, Voarino M, Barbati F, Moriondo M, Nieddu F, Sarli WM, Citera F, Astorino V, Pelosi C, Quaranta F, Stocco S, Canessa C, Lastrucci V, Ricci S, Indolfi G, Azzari C.Front Pharmacol. 2024 May 22;15:1381107. doi: 10.3389/fphar.2024.1381107. eCollection 2024.PMID: 38841370
2. Harris M, Clark J, Coote N, Fletcher P, Harnden A, McKean M, et al. British Thoracic Society guidelines for the management of community acquired pneumonia in children: update 2011. Thorax. 2011 Oct 1;66(Suppl 2):ii1–23.
