# Supplementary material for: Antibiotic optimization in hospitalized children with non-severe community-acquired pneumonia: lessons from an antimicrobial stewardship intervention (2022–2024)
Source: Front Pediatr. 2025 Nov 4;13:1660776. doi: 10.3389/fped.2025.1660776 (PMC12623335; doi:10.3389/fped.2025.1660776)
Supplement: Supplementary file 2 [file Datasheet1.pdf]

Diagnostic and therapeutic flow-chart for CAP in children (2 months - 18 years old)

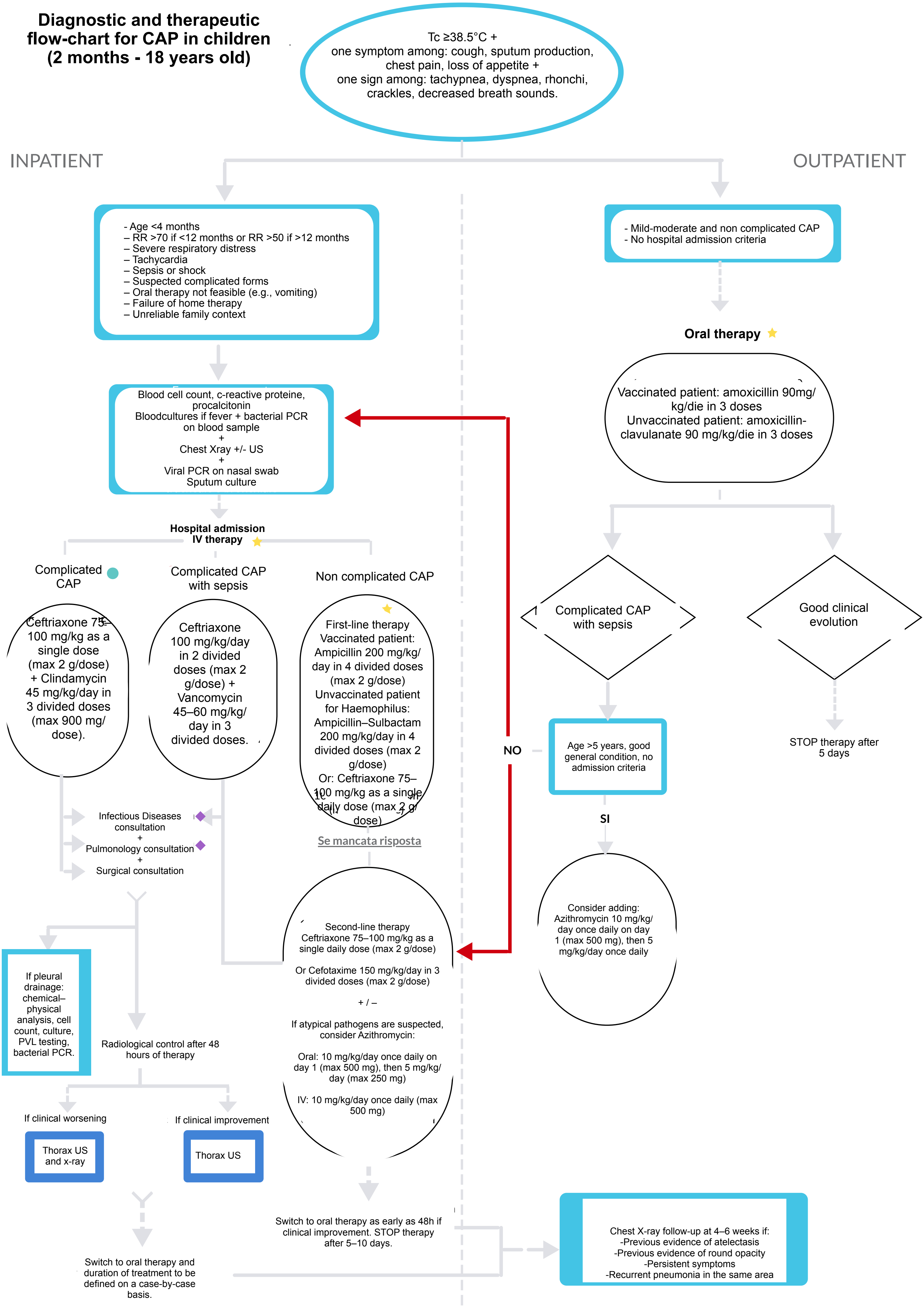

DEFINITION OF COMPLICATED CAP

Community-acquired pneumonia (CAP) is defined as complicated in the presence of local and/or systemic complications.

Local complications: pleural effusion, pleural empyema, pneumatocele, pneumothorax, broncho-pleural fistula, lung abscess, necrotizing pneumonia.

Systemic complications: bacteremia and metastatic infections, hyponatremia, sepsis, septic shock, multiorgan failure.

SEVERITY CRITERIA

Presence of at least one of the following signs/symptoms:

Respiratory failure requiring invasive or non-invasive mechanical ventilation, or high-flow nasal cannula (HFNC) with increasing FiO_2 requirements (>40%) or high flow rates (>2 L/kg/min or >40 L/min).

Systemic signs of inadequate perfusion (altered mental status, hemodynamic instability).

Respiratory rate (RR) above the age-specific upper limit.

Age	Upper limit (breaths/min)	Lower limit (breaths/min)
1 month	60	25
1 year	50	20
2 years	40	18
5 years	30	17
10 years	25	14

PCR on blood: Streptococcus pyogenes, Staphylococcus aureus, Streptococcus pneumoniae, Haemophilus influenzae, Adenovirus

PCR on nasopharyngeal swab: respiratory viruses PCR ± Mycoplasma pneumoniae and Chlamydia pneumoniae (based on clinical judgment)

PCR on pleural fluid: Streptococcus pneumoniae, Haemophilus influenzae, Streptococcus pyogenes, Staphylococcus aureus and 16S PCR (red-top tube)

THERAPY IN CASE OF PENICILLIN ALLERGY
Uncomplicated CAP – Oral therapy
Low-risk allergy: Cefpodoxime proxetil 10 mg/kg/day in 2 divided doses (max 400 mg/day)
High-risk allergy: Levofloxacin 16–20 mg/kg/day in 2 divided doses (max 750 mg/day)
or Clindamycin 30–40 mg/kg/day in 3–4 divided doses (max 600 mg/dose)
or Azithromycin 10 mg/kg/day once daily on day 1 (max 500 mg), then 5 mg/kg/day once daily (max 250 mg)

Uncomplicated CAP – Intravenous therapy
Low-risk allergy: Ceftriaxone 75–100 mg/kg as a single daily dose (max 2 g/dose)
or Cefotaxime 150 mg/kg/day in 3 divided doses (max 2 g/dose)
High-risk allergy: Levofloxacin 16–20 mg/kg/day in 2 divided doses (max 750 mg/day)
or Clindamycin 20–40 mg/kg/day in 3 divided doses (max 900 mg/dose)
or Azithromycin 10 mg/kg/day once daily (max 500 mg)

Complicated CAP – Intravenous therapy
Low-risk allergy: Ceftriaxone 75–100 mg/kg as a single daily dose (max 2 g/dose) + Clindamycin 45 mg/kg/day in 3 divided doses (max 900 mg/dose)
High-risk allergy: Levofloxacin 16–20 mg/kg/day in 2 divided doses (max 750 mg/day) + Clindamycin 45 mg/kg/day in 3 divided doses (max 900 mg/dose)

FURTHER INDICATIONS FOR SPECIALIST CONSULTATIONS
Infectious Diseases: positive blood culture, microbiological isolates from sputum, tracheal aspirate or drained material; complex patient; immunocompromised or with known previous colonization; history of persistent cough; contact with tuberculosis or coming from endemic areas.

Pulmonology: chronic pulmonary disease, recurrent pneumonia (>2 episodes/year).

Cardiology: underlying heart disease.

INFLUENZA PNEUMONIA

Treatment with Oseltamivir is indicated in all hospitalized patients with influenza infection or at high risk of complications due to underlying conditions (e.g., chronic disease, iatrogenic immunosuppression).

DISCHARGE CRITERIA
Improvement in general condition and respiratory dynamics.
Afebrile and SpO_2 >92% in room air for at least 24 hours.
Resumption of hydration and feeding.
Removal of any chest drainage tube for at least 24 hours, without evidence of clinical and/or radiological worsening.
Adequate family compliance.
